# Commentary: Rituximab combined with lenalidomide for the treatment of marginal zone lymphoma with IgM kappa positivity and cold agglutinin syndrome: a case report

**DOI:** 10.3389/fimmu.2026.1855684

**Published:** 2026-05-22

**Authors:** Zilong Wang, Chunhui Wang, Xiaobo Wang, Bo Tang

**Affiliations:** Department of Hematology, The Second Hospital of Dalian Medical University, Dalian, China

**Keywords:** case report, cold agglutinin syndrome, lenalidomide, marginal zone lymphoma, venous thromboembolism

## Introduction

We highly appreciate the case reported by Zhang et al., in which a patient with marginal zone lymphoma (MZL)-associated cold agglutinin syndrome (CAS) achieved complete remission using the R2 regimen (rituximab combined with lenalidomide) following no response to rituximab monotherapy (1). Based on this single case, the R2 regimen may serve as a useful reference for the treatment of MZL-associated CAS, while also demonstrating the potential value of lenalidomide as an immunomodulatory drug (IMiD) in indolent lymphomas.

## Immunomodulatory mechanisms and therapeutic implications of lenalidomide

The patient presented with prolonged peripheral cyanosis as the main manifestation. Hemolysis is often more urgent than indolent lymphoma itself, and monoclonal IgM should be suppressed quickly. Zhang et al. combined flow cytometry and molecular pathological testing to rule out the diagnosis of Waldenström macroglobulinemia (WM) based on the absence of both *MYD88* and *CXCR4* mutations. However, in the presence of systemic monoclonal IgM, WM should still be regarded as the top consideration in the differential diagnosis. In our clinical practice, we have also encountered occasional cases of MYD88 wild-type WM. These cases exhibit bone marrow morphology that overlaps with MZL and present with atypical clinical manifestations, falling into a diagnostic gray zone that makes differentiation challenging (2). It is worth noting that such patients often exhibit suboptimal responses to BTK inhibitors (3). Therefore, lenalidomide, as an IMiD, has become one of the options for overcoming drug resistance or for treating diseases within this diagnostic gray zone.

A potential mechanism contributing to the success of the R2 regimen in this case is lenalidomide’s hypothesized ability to overcome rituximab resistance. Lenalidomide not only exerts direct cytotoxic effects on clonal B cells by degrading IKZF1/3, but also remodels the immune function of T cells and natural killer (NK) cells, thereby enhancing antibody-dependent cellular cytotoxicity (ADCC) mediated by rituximab (4, 5). These dual synergistic mechanisms could explain the R2 regimen’s capacity to rapidly suppress monoclonal IgM and reduce cold agglutinin titers. However, lenalidomide exhibits heterogeneous immunomodulatory effects. Brauer et al. reported cases in which lenalidomide induce or even exacerbate cold agglutinin disease (CAD) in patients with myelodysplastic syndromes (MDS) (6). This suggests that the immunomodulatory impact of IMiDs may be opposite in different disease backgrounds.

## Thrombotic risk and safety considerations of lenalidomide

Despite the encouraging results reported, given the clear risk of venous thromboembolism (VTE) with lenalidomide, we believe it is necessary to evaluate its safety. Drawing a parallel to multiple myeloma (MM)—a malignancy similarly characterized by abnormal monoclonal immunoglobulin proliferation—patients treated with lenalidomide-based regimens without thromboprophylaxis have been reported to have a cumulative VTE incidence of up to 12% within the first 3 months (7). Although the VTE incidence in patients with indolent lymphoma using lenalidomide is slightly lower than in MM (8), the addition of erythrocyte agglutination and large amounts of monoclonal IgM from CAS forms the basis for endothelial damage and blood stasis, making the VTE risk with lenalidomide not to be underestimated. Especially for elderly patients in this case, the risk would be further increased if they also had obesity or infection.

However, there remains a lack of consensus regarding optimal strategies for the prophylaxis of IMiD-associated VTE, such as those induced by lenalidomide. A Cochrane evidence-based review concludes that the certainty of evidence from randomized controlled trials (RCTs) comparing the relative efficacy of aspirin, low-molecular-weight heparin (LMWH), and direct oral anticoagulants (DOACs, e.g., rivaroxaban) in preventing IMiD-associated thrombosis is “very low” (9). In the absence of a gold-standard approach, we believe that when administering the R2 regimen in such patients, it is advisable to implement personalized thromboprophylaxis management. This should be guided by incorporating patient-specific characteristics and adapting validated IMiD-associated VTE risk assessment models from the multiple myeloma field—namely, IMPEDE VTE score (10) and SAVED score (11). Following a comprehensive clinical evaluation, clinicians might consider the use of prophylactic agents such as low-dose aspirin or rivaroxaban, coupled with dynamic monitoring of coagulation profiles, to help mitigate the risk of adverse events.

## Discussion

The case presented by Zhang et al. provides a valuable reference for the management of refractory lymphoma-associated CAS, particularly highlighting the potential role of lenalidomide and other IMiDs in overcoming resistance to traditional monoclonal antibody therapy. Looking ahead, we anticipate further research focusing on precision subtyping within the diagnostic gray zone between MZL and WM. Concurrently, it is imperative to establish and optimize standardized safety management guidelines for the use of lenalidomide and other IMiDs in patients with underlying hypercoagulable states.
